# A Closed-Loop Clinical Audit of Surgical Documentation of Inpatient Records at a Tertiary Level Hospital in Egypt

**DOI:** 10.7759/cureus.49862

**Published:** 2023-12-03

**Authors:** Ahmed G Hassan, Eyad O Elqaffas, Ahmed M Elbouridy, Mazen M Shawky, Tarek A El-Fayoumi

**Affiliations:** 1 General Surgery Department, Faculty of Medicine, Alexandria University, Alexandria, EGY; 2 Anesthesiology Department, Alexandria Main University Hospital, Alexandria, EGY; 3 Surgical Oncology Department, Alexandria Main University Hospital, Alexandria, EGY

**Keywords:** record keeping, quality improvement, alexandria main university hospital, star score, clinical audit, surgical records

## Abstract

Background: Optimal record keeping is a very essential component in health care provision especially in the surgical setting. This study aimed to evaluate the quality of surgical records in wards of a surgical department at Alexandria Main University Hospital, Egypt.

Methods: We created a systematically designed checklist using standard hospital protocol and universal guidelines presented in the previously validated STAR and CRABEL auditing tools as a basis for Yes/No questions. This checklist was then used to prospectively evaluate the quality of surgical records of patients who underwent surgery in the surgical oncology department from July 2023 to October 2023. Total STAR and section-specific STAR scores were then calculated and compared statistically.

Results: A total of 80 records were randomly selected and evaluated using the STAR questionnaire. All domains showed improvement compared to the baseline except for the discharge summary which did not change from an already relatively high baseline of 96±0.0. The highest improvements were observed in the anesthetic record and operative record domains which increased from 90.65±4.3 and 86.15±5.347 to 100±0.0 and 95.6±3.365, respectively.

Conclusion: Our study demonstrates that significant improvements in the quality of surgical records can be achieved by simply using preprepared templates, personnel education, and systematic auditing.

## Introduction

Medical records are essential for healthcare professionals to plan and evaluate patients’ treatment, ensuring continuity of care across providers [1]. The accuracy, readability, and thoroughness of these records are crucial for maintaining a high standard of patient care that meets the certification requirements and universal guidelines [2]. We should strive for documentation that strikes a balance between being both comprehensive and efficient, delivering valuable information without impeding proper care through unnecessary time and resource consumption [3].

Effective record-keeping is particularly crucial in surgical settings due to the high stakes involved in major interventions and the management of critical patients. This thereby minimizes the margin for error, making optimal record-keeping paramount. When done correctly, it offers a comprehensive overview of both major and minor details related to the patient's case, benefiting both the patient and the surgeon. For the patient, thorough record-keeping ensures that all pertinent information is readily available. This facilitates continuity of care, as healthcare providers can easily access and review the patient's medical history, surgical procedures performed, medications administered, and other relevant data. Having access to accurate and up-to-date records enables healthcare professionals to make well-informed decisions, tailor treatment plans, and provide appropriate follow-up care. In addition, optimal record-keeping in surgery promotes better coordination among healthcare professionals involved in the patient's treatment. Accurate and complete records can be shared among different members of the healthcare team, including surgeons, anesthesiologists, nurses, and other specialists. This sharing of information enhances communication, collaboration, and teamwork, leading to more coordinated and effective patient care. This ultimately contributes to the overall standard of healthcare services provided to the patient [4].

Furthermore, maintaining a complete record serves as a protective measure for the surgeon in potential legal disputes. In the unfortunate event of litigation or claims, a well-documented record can provide crucial evidence. It can demonstrate that the surgeon adhered to established protocols, guidelines, and best practices in the field. Moreover, it can provide a clear account of the rationale behind specific interventions or treatments performed by the surgeon. This documentation can be instrumental in defending the surgeon's actions and mitigating potential legal risks [5]. Therefore, optimal record-keeping in surgery plays a vital role in enhancing patient care, ensuring the highest standards of medical practice, and safeguarding the interests of both patients and surgeons.

Clinical audit is a quality-improvement process that involves reviewing all aspects of medical practice and comparing it to predetermined guidelines to assess the quality of care provided and improve treatment. The insights gained from this process can be used to identify areas of deficit where there could be room for improvement in patient care by adjusting practices as needed [6]. The National Institute of Clinical Excellence (NICE) defines clinical audit as “A quality improvement process that seeks to improve patient care and outcomes through systematic review of care against explicit criteria and the implementation of change” [7].

A variety of tools have been created to assess the quality of clinical records. One such tool is the CRawford, BEresford, Lafferty (CRABEL) scoring system, developed by CRAwford- BEresford-Lafferty, which can be utilized in any in-patient specialty [8]. Another tool called the Surgical Tool for Auditing Records (STAR) is a modified version of the CRABEL system specifically designed for auditing surgical records. The STAR score has been validated through its application in numerous studies conducted across different departments and institutions. These studies have consistently demonstrated positive improvements, confirming the effectiveness of the STAR score [9-13].

The aim of our study is to assess whether the current documentation procedure complies with hospital protocol and meets the criteria of CRABEL [8] and STAR auditing tools [9]. Moreover, it aims to identify areas of deficit in the documentation process relative to the guidelines and to raise the standard of clinical records kept by surgical staff, with the goal of enhancing the quality of care and treatment provided to patients.

## Materials and methods

Study design

This is a prospective, quantitative, descriptive study.

Study area

Wards of Surgical Oncology Department in Alexandria Main University Hospital, Egypt.

Sample size

A methodical approach was employed to randomly select and evaluate a subset of inpatient medical records from wards of the Surgical Oncology Department during the time period between July 21, 2023 and October 3, 2023. A sample of 80 random cases was selected for our study, with an equal distribution of 40 cases in both the first and second cycles. The 80 cases included ones referred from the surgical oncology clinic and the emergency department, as well as cases referred to the intensive care unit. These randomly sampled records were then examined to determine the adequacy of documentation and record keeping.

Data collection

Inpatient records in the surgical oncology ward were manually reviewed and subsequently scored individually using the STAR. The STAR system, which has a high level of reliability (Cronbach's α: 0.959), serves as a dependable alternative to CRABEL for medical record keeping. It consists of 50 components divided into six domains with varying weight allocations: Initial Clerking (10 items; 20%), Subsequent Entries (eight items; 16%), Consent (seven items; 14%), Anesthetic Record (seven items; 14%), Operative Record (nine items; 18%), and Discharge Summary (nine items; 18%). To utilize the tool, a minimum of 20 records is required. The total score for each assessed note is determined using the formula: (50 - deducted points) x 2. The overall STAR score is calculated as the average of all evaluated notes [9]. Data collection was conducted regularly, twice a week, at a fixed time: noon on the first postoperative day. This scheduling aimed to standardize the time factor and its impact on the completeness of surgical records.

Interventions

Multiple specific interventions to improve the quality of surgical record-keeping ran concurrently between the first and second audit cycles. Templates for postoperative reports, postoperative anesthesia reports, and daily follow-up sheets were created based on the STAR guidelines and hospital protocols. These templates were provided to residents in the surgical oncology department and the anesthesiology department.

Efforts were made to establish coordination between the surgical oncology department and the anesthesiology department. Senior residents in the anesthesiology department were informed about the ongoing clinical audit and the deficiencies identified in the postoperative anesthesia reports during cycle one. They were also made aware of the newly designed postoperative anesthesia report template and were given the opportunity to suggest any changes they deemed necessary. Once an agreement was reached, they were encouraged to complete and submit the postoperative anesthesia reports within 24 hours after the surgery, with an emphasis on the importance of timely paperwork.

The nursing staff received education on the identified issues with the surgical records, including the potential loss of important surgical documents and the legal implications associated with it. They were also introduced to the new templates and instructed to ensure their timely completion and delivery.

Surgical oncology residents were informed about the results of cycle one of the clinical audits and were reminded about various aspects of record-keeping that may have been overlooked. These aspects included accurately recording the dates on surgical consent forms and inquiring about and documenting drug allergies during patient history-taking. Furthermore, they were provided with the operative report and patient daily follow-up templates and were encouraged to incorporate them into their routine patient care and consistently fill them out and keep them in the patient's record.

Statistical analysis

The collected data were reported as frequencies (n (%)). Pre- and post-audit total STAR scores, as well as section-specific STAR scores, were calculated and presented as means ± standard deviations. A comparison was made between the pre- and post-audit scores, and the percentage increase or decrease was calculated. All data cleaning and statistical analyses were performed using SPSS Statistics, version 23 (IBM Corp., Armonk, NY). The study protocol was reviewed and approved by the Alexandria University research ethics committee. The procedures adhered to the guidelines of the Declaration of Helsinki (1996).

## Results

**Figure 1:**
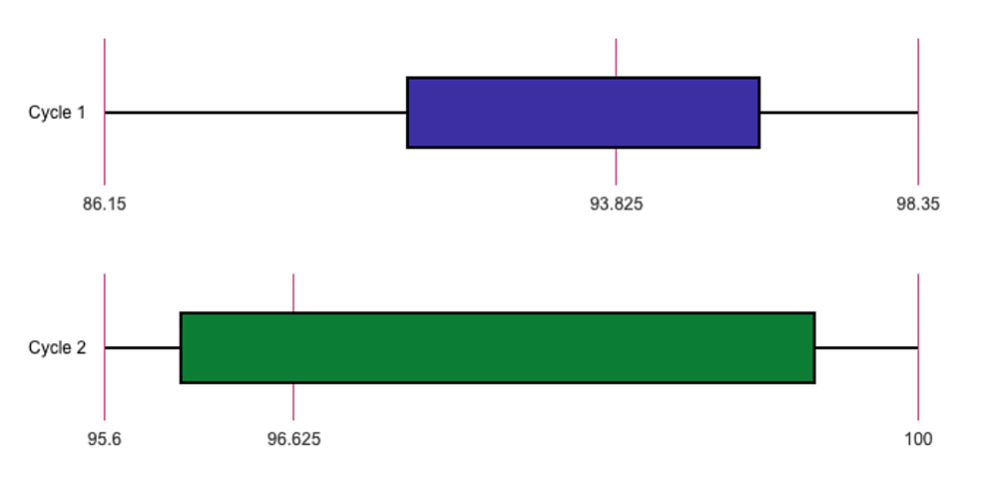
Box plots for total STAR scores for cycles one and two STAR - Surgical Tool for Auditing Records

Between July 21, 2023 and October 3, 2023, a total of 80 surgical records of patients undergoing surgery in the surgical oncology department were evaluated using the STAR scoring system. The results, presented in Table 1, show the total STAR scores and section-specific STAR scores and percentage increase for Cycle one and Cycle two. Overall, there was an increase in the total score of the surgical records from 93.13 in Cycle one to 97.38 in Cycle two, indicating the effectiveness of the proposed solutions and the clinical audit (Figure 1).

With the exception of the discharge summary, which remained unchanged at a relatively high baseline of 96%, all domains of the study demonstrated varying levels of improvement from Cycle one to Cycle two. The anesthetic record and operative record domains exhibited the greatest enhancements, while the subsequent entries domain showed the least improvement, although this can be attributed to the already high pre-audit baseline (Table 1).

**Table 1:** Total STAR scores, section-specific STAR scores, and percentage increase for cycles one and two STAR - Surgical Tool for Auditing Records

Criteria	STAR score cycle 1	STAR score cycle 2	Percentage Increase
Initial Clerking	93.65±0.893	97.25±0.98	3.844
Subsequent Entries	98.35±0.8876	99.45±0.904	1.12
Consent	94±0.0	96±0.0	2.13
Anesthetic Record	90.65±4.3	100±0.0	10.314
Operative Record	86.15±5.347	95.6±3.365	10.97
Discharge Summary	96±0.0	96±0.0	0
Total Score	93.13±4.275	97.38±1.9	4.56

In the initial clerking domain, the mean score increased from 93.65 in Cycle one to 97.25 in Cycle two. The subdomain that showed the most significant improvement was the referral source, which had no recorded instances in Cycle one but achieved a 100% recording rate in Cycle two. Other subdomains, such as working diagnosis and investigations/results, also improved from 85% and 97.5% recording rates to 100% in both cases. Allergies recorded increased substantially from 0% in Cycle one to 62.5% in Cycle two. The remaining subdomains showed no change between the cycles, primarily due to a perfect recording rate of 100% in Cycle one.

**Figure 2:**
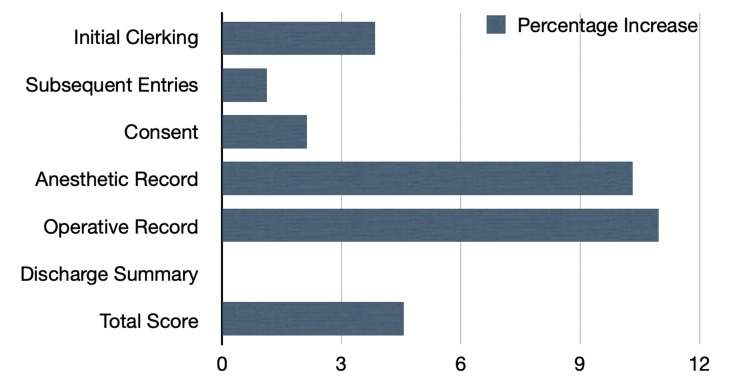
Percentage Increase in STAR scores from cycle one to cycle two

The subsequent entries domain experienced a slight 1.12% increase (Figure 2), largely due to its high pre-audit mean score of 98.35. Most subdomains either showed a slight increase or remained at a perfect recording percentage, except for relevant comments on patient state and examination appropriate to the level of the case, which increased from eight cases to 29 cases out of the 40 assessed records in each cycle.

In the consent domain, there was an improvement from 94 to 96. The name/number/date subdomain was solely responsible for this increase, with a change from zero cases recorded in Cycle one to 40 cases in Cycle two. The remaining subdomains showed no change.

**Figure 3:**
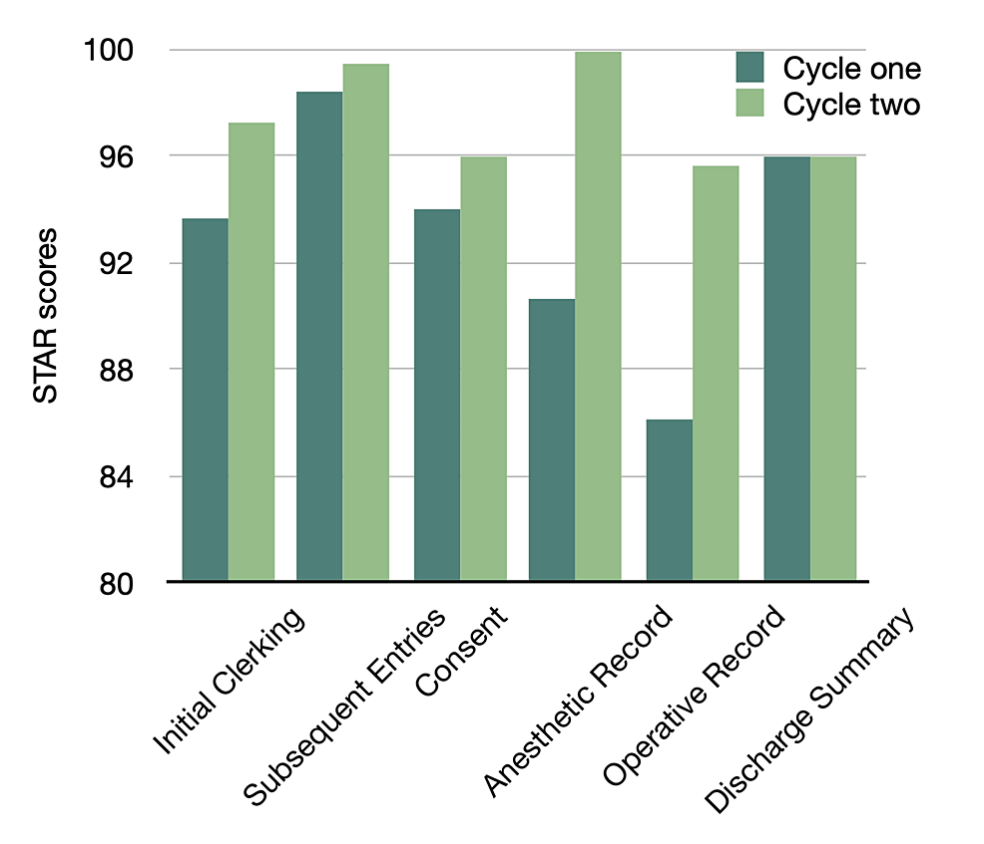
Section-specific STAR scores for cycles one and two

As shown in Table 1 and Figure 3, the anesthetic record was the only domain to reach a perfect score of 100 in Cycle two, although the baseline was a modest 90.65. The pre-operative assessment showed no defects in both cycles. However, significant deficiencies were observed in other subdomains in Cycle one. The recording rates for drugs and doses given during anesthesia and monitoring data were 7.5% and 20%, respectively. The IVI given and name/signature subdomains had a recording rate of 30%. Post-anesthetic instructions were present in 15% of the records, and the name of the anesthetist/consultant was recorded in 25% of the cases. However, all of these subdomains showed substantial improvements, reaching 100% in Cycle two, making the anesthetic record the most efficiently recorded domain in the surgical records.

The operative record domain exhibited the highest percentage increase, with an improvement of 10.97%. This domain had the lowest baseline of 86.15 and reached 95.6 after the interventions implemented between the cycles. All subdomains within this domain showed significant improvements, except for prosthetics/serial numbers and postoperative instructions. Noteworthy enhancements included the operating surgeon's name and details of sutures used, which both increased from 0% to 65%. A comprehensive overview of the results of all the aforementioned subdomains is presented in Table 2.

**Table 2:** Detailed results and percentages for cycles one and two

Domain	Subdomains	Cycle 1	Cycle 2
Initial Clerking	Name	40 (100%)	40 (100%)
	Hospital number	40 (100%)	40 (100%)
	Referral Source	0 (0%)	40 (100%)
	Consultant	0 (0%)	0 (0%)
	Date/Time	40 (100%)	40 (100%)
	Working diagnosis	34 (85%)	40 (100%)
	Investigations/Results	39 (97.5%)	40 (100%)
	Management plan	40 (100%)	40 (100%)
	Allergies Recorded	0 (0%)	25 (62.5%)
	Name/Bleep/Post	40 (100%)	40 (100%)
Subsequent Entries	Name/Number	40 (100%)	40 (100%)
	Date/Time	40 (100%)	40 (100%)
	Heading	40 (100%)	40 (100%)
	Relevant comment on patient state and examination appropriate to level of case	8 (20%)	29 (72.5)
	Pertinent results	39 (97.5%)	40 (100%)
	Plan	40 (100%)	40 (100%)
	Signature/Name/Bleep/Post	40 (100%)	40 (100%)
	Legibility	40 (100%)	40 (100%)
Consent	Name/Number/Date	0 (0%)	40 (100%)
	Operation	40 (100%)	40 (100%)
	Side and site in full words	40 (100%)	40 (100%)
	Benefits	0 (0%)	0 (0%)
	Risks/Complications	40 (100%)	40 (100%)
	Signatures	40 (100%)	40 (100%)
	Name/Bleep/Post	0 (0%)	0 (0%)
Anesthetic Record	Name of anesthetist/consultant	10 (25%)	40 (100%)
	Pre op assessment	40 (100%)	40 (100%)
	Drugs and doses given during anesthesia	3 (7.5%)	40 (100%)
	Monitoring data	8 (20%)	40 (100%)
	IVI given	12 (30%)	40 (100%)
	Post anesthetic instructions	6 (15%)	40 (100%)
	Name/Signature	12 (30%)	40 (100%)
Operative Record	Name/Number/Date	12 (30%)	34 (85%)
	Operating surgeon	0 (0%)	26 (65%)
	Diagnosis post prcedure	14 (35%)	26 (65%)
	Description of findings	8 (20%)	40 (100%)
	Details of tissues removed	13 (32.5%)	40 (100%)
	Details of sutures used	0 (0%)	26 (65%)
	Prosthetics/serial numbers	0 (0%)	0 (0%)
	Post operative instructions	40 (100%)	40 (100%)
	Surgeon/Signature	12 (30%)	40 (100%)
Discharge Summary	Name/Number/address	40 (100%)	40 (100%)
	Admission/Discharge dates	40 (100%)	40 (100%)
	Discharging consultant	0 (0%)	0 (0%)
	Diagnosis	40 (100%)	40 (100%)
	Pertinent investigations/results	0 (0%)	0 (0%)
	Operations/procedures	40 (100%)	40 (100%)
	Complications	40 (100%)	40 (100%)
	Medication on discharge	40 (100%)	40 (100%)
	Follow-up	40 (100%)	40 (100%)

## Discussion

The rationale of our quality improvement study was to evaluate how well the STAR checklist was being followed in our current surgical setting. The goal was to develop an efficient plan to improve adherence to the STAR checklist and enhance the ongoing provision of patient care [14]. It should be acknowledged that the reason for the relatively limited number of samples was due to the discovery, during data collection, that the defects were repetitive and followed a pattern, specifically, the same parameters consistently exhibited the same defects. This led us to suspect a lack of awareness or potential negligence regarding the guidelines for maintaining surgical records. As a result, we determined that a sample size of 40 records would be sufficient, and there was no need to invest additional time before implementing changes to address the significant defects [15]. However, it is recommended to conduct further cycles in the future with a larger sample size to identify any minor defects that may have been overlooked.

The STAR scoring system, an auditing tool used to assess the quality of medical record-keeping, was employed in this study to evaluate the surgical records in the surgical oncology department of Alexandria Main University Hospital. The audit covered a period of approximately 2.5 months and included a total of 80 cases. The results, as shown in Table 1, indicated improvements in nearly all aspects of the surgical record, leading to a high mean total STAR score of 97.38±1.9. These improvements were statistically significant when compared to the baseline score of Cycle one. The findings of this study align with previous literature on the STAR scoring system. Tuffaha et al. documented a rise in the overall STAR score from 83.4 to 97.6 following the completion of an audit cycle [9]. Similarly, Basu et al. observed an enhancement in the STAR score from 87 in the initial cycle to 93 in the subsequent cycle [10]. Additionally, Alqudah et al. observed a notable progression in the total STAR scores over the years. Starting from a baseline of 95.2±1.2 in 2016, they achieved the highest score of 97.3±0.9 in 2021 [11]. Chalikonda et al. also observed an improvement in the STAR score from 76.7 to 81 over a span of 10 years [12]. However, it is important to note that the relative improvement in the surgical oncology department of Alexandria Main University Hospital was lower compared to these previous audits. This could be attributed to the higher baseline STAR score in the surgical oncology department, which started at 93.13±4.275.

**Figure 4:**
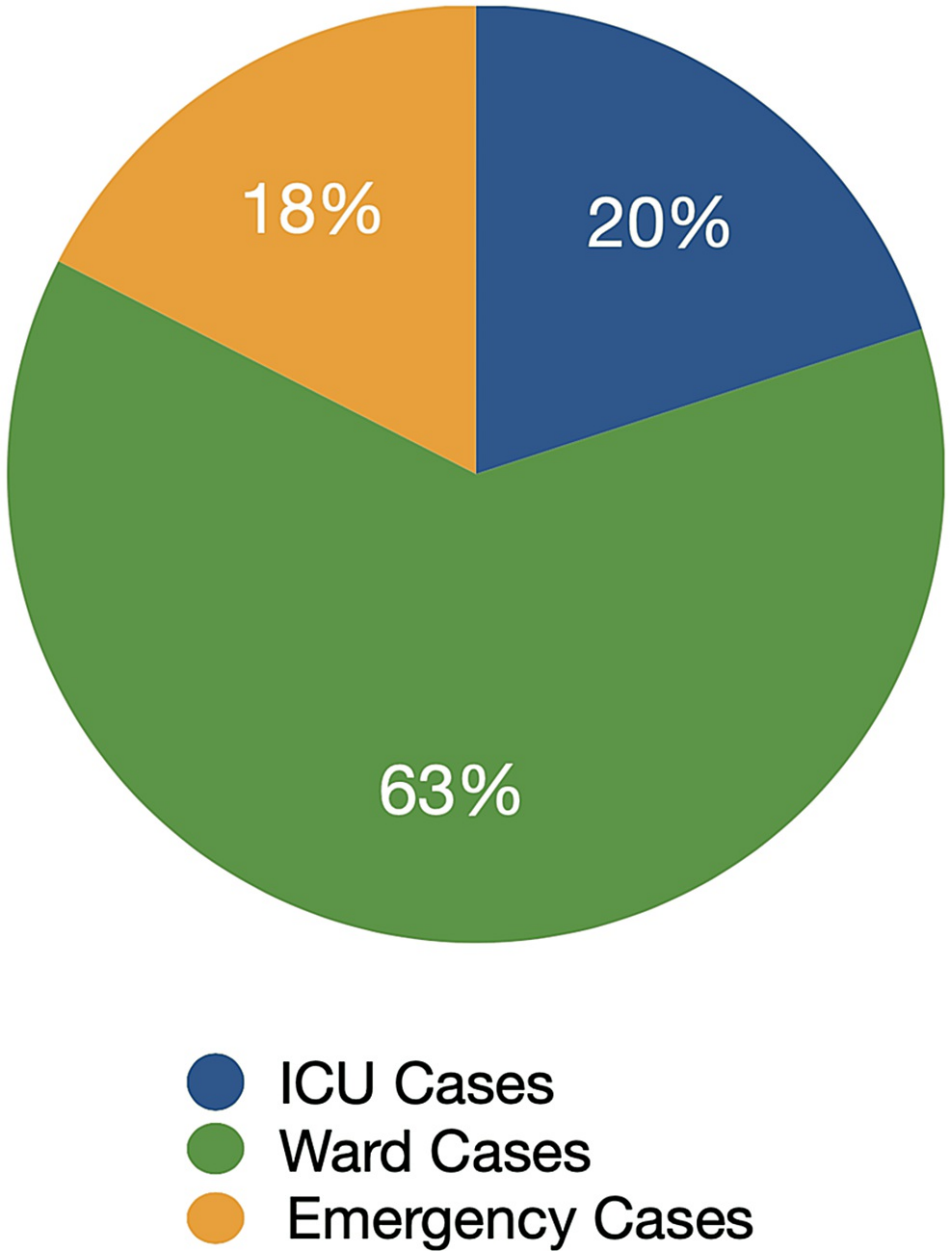
Stratification of assessed cases in cycle one

**Figure 5:**
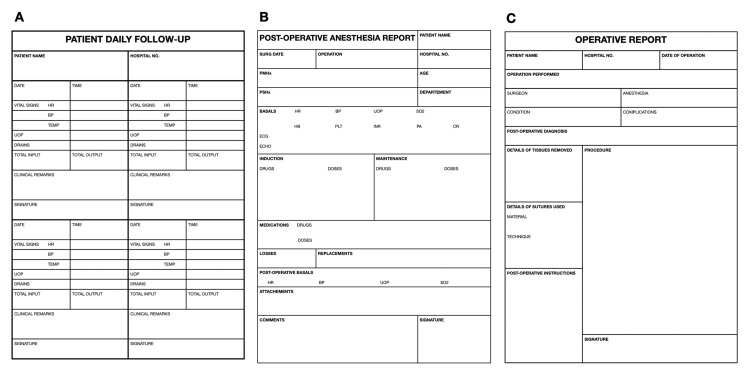
(A) Patient daily follow-up template, (b) postoperative anesthesia report template, and (c) operative report template

During Audit Cycle one, we examined 40 records, which included different types of cases. Out of these, eight cases were referred to the intensive care unit (ICU) after their operations, 25 cases were referred from the surgical oncology clinic and stayed in the department wards postoperatively, and seven cases were referred from the emergency department (Figure 4). This distinction among cases proved to be significant because certain checklist items were recorded differently depending on the referral source and destination. Notably, all ICU cases had relevant comments on the patient state and examination appropriate to the level of the case as well as input and output charts in their records, and there was a 100% compliance rate in the subsequent entries subdomain. However, deficiencies were observed in this subdomain for cases in the department wards. To address this issue, we developed a daily follow-up sheet template (Figure 5A) and provided it to surgical oncology residents. This template aimed to provide a structured approach to patient management, promote systematic care delivery, facilitate data recording and sharing, and ensure adherence to evidence-based guidelines.

The effectiveness of using templates in record-keeping was previously described by Morrissey et al. in 2021 [16], who defined templates as various forms, checklists, questionnaires, proformas, or smart forms that support structured patient management and enable high-quality care delivery. The use of templates can be a valuable tool in enhancing record-keeping practices, as they provide a standardized framework for documenting essential information and ensure that key elements are not overlooked. Furthermore, templates can contribute to the aggregation of data, which can be utilized to assess institutional performance and identify areas for improvement. In the current study, the results of Cycle two demonstrated the effectiveness of templates in improving record-keeping. Specifically, the compliance rates for relevant comments on the patient's state and examination to the level of the case increased from 20% in Cycle one to 72.5% in Cycle two. This substantial improvement suggests that the introduction of the daily follow-up sheet template positively influenced the documentation of relevant patient information and examinations.

During Cycle one of the audit, a concerning finding was made regarding the recording of drug allergies in the surgical records. None of the 40 cases in Cycle one had drug allergies documented, which posed a potential risk to patient safety. Overlooking drug allergies, which could be known by the patient, can lead to severe consequences, including life-threatening anaphylaxis, that could have been easily prevented. The failure to record drug allergies not only puts patients' health at risk but also exposes surgeons to potential medico-legal disputes [17]. To address this issue, a reminder was given to the surgical team about the importance of inquiring about drug allergies and documenting them accurately. In Cycle two, there was a significant improvement, with 25 out of the 40 records documenting drug allergies or the absence thereof. This contrasted with Cycle one, where none of the records included this information. Although there is still room for further improvement, this progress represents a step in the right direction.

**Figure 6:**
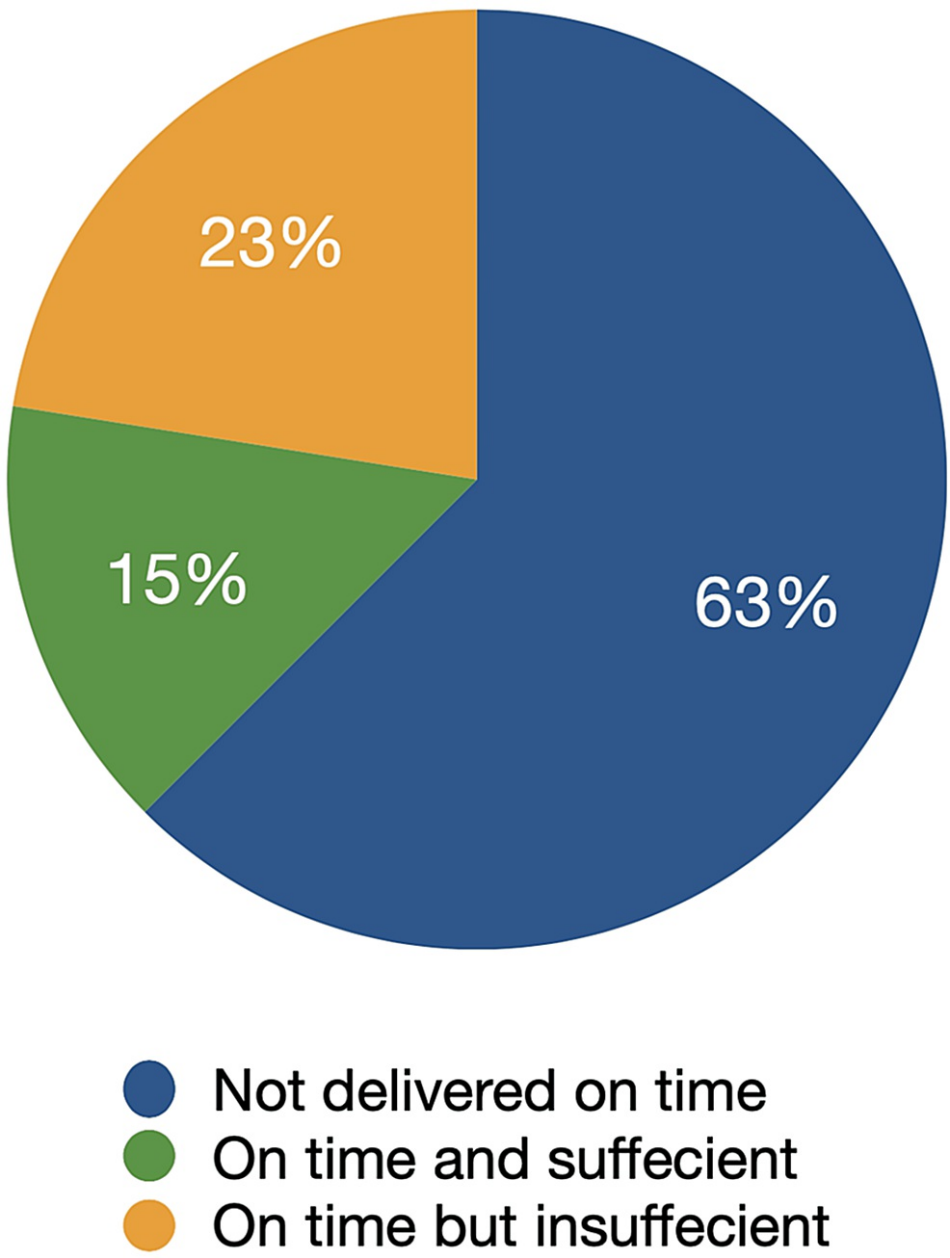
Postoperative anesthesia report stratification in cycle one

The most noteworthy defects in surgical record keeping noted during cycle one were concerning the anesthesia record and the operative record. Two major issues were observed: the delay in delivering these reports and their lack of completeness according to international guidelines and hospital protocols. Data collection for the audit was conducted on the first postoperative day, approximately 24 hours after the surgeries. However, 25 postoperative anesthesia reports and 24 operative reports were not provided at all. This may be attributed to a lack of awareness among the staff regarding the importance of timely paperwork delivery and optimal record keeping [18]. Additionally, the scarcity of recognition concerning the purpose and both legal and medical benefits of such reports among the surgery and anesthesia staff contributes to its poor conformity. In cases where the reports were delivered on time, a significant proportion of them were substandard and deviated from the guidelines. Only three postoperative anesthesia reports were both complete and delivered on time, while no operative reports met both criteria (Figure 6). To address the shortcomings identified, a two-step approach was taken. The first step involved creating templates similar to the aforementioned daily follow-up template for both postoperative anesthesia reports (Figure 5B) and operative reports (Figure 5C). These templates ensured that the guidelines were followed and encouraged comprehensive documentation of the required information, in line with the findings of Morrissey et al. in 2021 [16]. The second step involved reminding surgical and anesthesia residents about the importance of timely submission of paperwork and advising them to submit these reports within 24 hours after the operation. Surprisingly, this approach was highly successful and led to a significant improvement in the quality of operative and anesthesia records in the second cycle. Quantitatively, the STAR scores for operative and anesthesia records increased from 86.15±5.347 and 90.65±4.3 to 95.6±3.365 and 100±0.0, respectively (Table 1).

A logical next step to further enhance the surgical records would be to implement a designated electronic record entry system, as it has been statistically proven to improve the overall quality and durability of records. The impact of such an intervention was demonstrated by Alqudah et al. in 2022, where they reported that the surgical records at JUH achieved a total STAR score of 97.3±0.9 in 2021, compared to a baseline of 95.2±1.2 in 2016, after implementing a designated electronic record entry system [11].

Although the STAR tool has demonstrated reliability and validity, it may not capture all the variables that are important to surgical patients. The STAR score has a specific structure that has its own limitations, as it may fail to identify major areas of deficiencies that are concealed within the vague nature of its concepts, without focusing on specific details. However, our study's strengths lie in our rigorous methodology. We conducted a survey of a random sample of medical records, which represents an average number of cases. All the cases in both cycles had similar levels of complexity, ensuring that we could exclude any variables that could impact our controlled study. We utilized a well-validated tool from the existing literature, which demonstrates excellent reliability and low variation among different observers [11,13].

Recommendations for future practice

It is essential to provide training to all surgical personnel on the universal guidelines for proper documentation practices and accurate completion of required documentation. Emphasizing the importance of maintaining records for medico-legal purposes is crucial for employees and nursing staff to understand and appreciate.

Efforts should be made to establish effective coordination between surgical departments and the anesthesiology department, particularly regarding the completeness and timely delivery of postoperative anesthesia reports. This can be achieved through initiatives that promote collaboration and communication between the departments.

To ensure compliance with guidelines, it is advisable to consistently provide pre-prepared blank templates for postoperative anesthesia reports, operative reports, and daily follow-up sheets to anesthesiology and surgical residents. This will assist in generating reports that align with the required standards. Allocating funds for printing these templates throughout the year for all surgical departments is necessary.

The results and proposed solutions from this clinical audit should be presented by the quality assurance unit to all surgical departments, enabling them to strive for the same high standards achieved by the surgical oncology department.

Regular assessment and evaluation of surgical records across all departments should be conducted by the quality assurance unit through clinical audits and quality assurance programs. A dedicated division should be formed with the objective of developing a database for the clinical audits’ outcomes. This ensures ongoing monitoring of the records' standard and accuracy.

Further clinical audits should be conducted in the surgical oncology department to assess the sustainability of the proposed solutions and to determine the medical and legal benefits attained from these interventions. Exploring the possibility of implementing a specified electronic record entry system is worth considering. A cost-benefit analysis should be conducted in the context of the Alexandria Main University Hospital to evaluate the feasibility of such a system.

## Conclusions

Indeed, surgical records can sometimes have significant deficiencies that can impact the quality of healthcare delivery, even in tertiary care hospitals. However, the implementation of templates for essential medical and surgical documents, along with education for medical personnel on the importance of record-keeping and proper practices, has demonstrated a notable improvement in the quality of surgical records, as evidenced by the improved STAR scores mentioned earlier. To ensure the sustainability of these improvements and to assess the ongoing adherence to the proposed solutions, further clinical audits should be conducted. These audits will help evaluate whether the recommended practices are consistently followed and will also provide insights into the medical and legal benefits derived from these interventions. By conducting regular audits, healthcare providers can continue to identify areas for improvement and make necessary adjustments to maintain and enhance the quality of surgical records.
